# IDENTIFYING EFFORTS AND CHALLENGES OF MARKETING THE DEPARTMENT OF VETERANS AFFAIRS MEDICAL FOSTER HOME PROGRAM

**DOI:** 10.1093/geroni/igad104.2850

**Published:** 2023-12-21

**Authors:** Leah Haverhals, Chelsea Manheim, Mary Nunnery, Cari Levy

**Affiliations:** Department of Veterans Affairs Rocky Mountain Regional VA Medical Center, Denver, Colorado, United States; Rocky Mountain Regional VAMC, Aurora, Colorado, United States; Rocky Mountain Regional VAMC, Aurora, Colorado, United States; University of Colorado, Denver, Colorado, United States

## Abstract

The United States (US) Department of Veterans Affairs (VA) Medical Foster Home (MFH) program is a long-term care (LTC) option for Veterans needing nursing home-level care. In the MFH program, Veterans live in the home of a community-based caregiver who has been recruited and screened by VA staff. Since 2016, VA Offices of Rural Health and Geriatrics and Extended Care has expanded the program, and by 2026 the VA plans to have programs at every VA Medical Center. To describe current efforts to market the MFH program, we conducted a national survey of MFH staff between Dec. 2, 2021-Jan. 6, 2022. We sent invitations via email, including a website link to the survey. N=94 completed the survey. Three team members analyzed open-ended data, using an inductive and deductive approach to thematic analysis. Findings revealed that external marketing strategies primarily focused on recruiting appropriate caregivers. Efforts included in-person presentations to community groups, utilizing local media outlets, manning booths at local health and job fairs, and providing written materials about the program. Internal marketing efforts included approaching many different VA departments including inpatient social workers, case managers, and discharge planners to identify Veterans for the program, and holding in-service presentations. Challenges included MFH coordinators having minimal time to market the program and receiving inconsistent support from the VA Public Affairs Office depending on location. Findings will inform expansion of the MFH program, which is paramount as the demand for LTC increases concurrently with the aging Veteran population.

